# Repurposing HIV Protease Inhibitors Atazanavir and Darunavir as Antifungal Treatments against *Candida albicans* Infections: An In Vitro and In Vivo Study

**DOI:** 10.3390/cimb44110364

**Published:** 2022-11-01

**Authors:** Juliana de C. Fenley, Patrícia P. de Barros, Paulo H. F. do Carmo, Maíra T. Garcia, Rodnei D. Rossoni, Juliana C. Junqueira

**Affiliations:** 1 Department of Biosciences and Oral Diagnosis, Institute of Science and Technology, São Paulo State University (Unesp), São José dos Campos, São Paulo 12245-000, Brazil; 2 Multicampi School of Medical Sciences, Federal University of Rio Grande do Norte (UFRN), Caicó, Rio Grande do Norte 59300-000, Brazil

**Keywords:** *Candida albicans*, virulence factors, HIV, protease inhibitors, atazanavir sulfate, darunavir

## Abstract

*Candida albicans* is the chief etiological agent of candidiasis, a mycosis prevalent in individuals with acquired immunodeficiency syndrome (AIDS). In recent years, the introduction of human immunodeficiency virus (HIV) protease inhibitors (HIV-PI) has reduced the prevalence of candidiasis in these patients. Seeking new therapeutic strategies based on the perspective of drug repositioning, we evaluated the effects of two second-generation HIV-PIs, atazanavir (ATV) and darunavir (DRV), on virulence factors of *C. albicans* and experimental candidiasis. For this, clinical strains of *C. albicans* were subjected to in vitro and in vivo treatments with ATV or DRV. As a result, ATV and DRV exhibited antifungal activity against fungal cells at 512 μg/mL, reduced the viability and biomass of biofilms, and inhibited filamentation of *C. albicans*. In addition, these HIV-PIs downregulated the expression of *SAP2* and *BRC1* genes of *C. albicans*. In an in vivo study, prophylactic use of ATV and DRV prolonged the survival rate of *Galleria mellonella* larvae infected with *C. albicans*. Therefore, ATV and DRV showed activity against *C. albicans* by reducing cell growth, biofilm formation, filamentation, and expression of virulence genes. Furthermore, ATV and DRV decreased experimental candidiasis, suggesting the repurposing of HIV-PIs as antifungal treatments for *C. albicans* infections.

## 1. Introduction

*Candida albicans* is an opportunistic fungus that usually colonizes the oral cavity, skin, gastrointestinal, and reproductive tracts [1]. Although it is harmless in healthy individuals, *C. albicans* can assume a pathogenic feature with rapid proliferation and invasion in the tissues, causing a variety of diseases. *C. albicans* is the most common causative agent of oral, vaginal, and disseminated candidiasis [2]. The annual incidence of *Candida* infections is about 4 million cases worldwide, involving an average healthcare cost of USD 65,000.00 per patient [3,4]. Furthermore, candidiasis is frequent among immunodeficient individuals, with oral candidiasis indicative of untreated human immunodeficiency virus (HIV) infection [5]. However, since the introduction of the first generation of HIV protease inhibitors (HIV-PI) in highly active antiretroviral therapy (HAART), the prevalence of candidiasis in HIV-infected individuals has reduced [6].

Despite the initial controversy about the role of HIV-PIs in decreasing the prevalence of candidiasis in HIV-infected individuals, clinical studies have demonstrated that HIV-PIs can exert antifungal activity against *C. albicans* associated with an improvement in the patient’s immune status [2,7]. It has been suggested that HIV-PIs can influence the virulence factors of *C. albicans*, including adhesion [8], morphogenesis [9], ability to form biofilm [10,11], and secretion of extracellular hydrolytic enzymes, especially the secreted aspartyl protease (Saps) [10,12].

Currently, the major classes of antifungal drugs used to treat *C. albicans* infections are polyenes, azoles, and echinocandins. However, these treatments exhibit limitations related to toxicity, cost, and antifungal resistance, especially to azole [13]. In the search for new therapeutic strategies based on drug repositioning, the use of HIV-PIs has become an interesting approach to the treatment of candidiasis [14]. The HIV-PIs inhibit the activity of HIV-1 protease, an enzyme that cleaves viral polyproteins during the virus maturation process [15,16]. The antifungal activity of first-generation HIV-PIs, such as saquinavir, ritonavir, and amprenavir, has been demonstrated in previous studies focusing on *Aspergillus candidus* [17], *Cryptococcus gattii*, *Cryptococcus neoformans* [18], and *C. albicans* [10,12]. However, the accumulation of drug-resistance mutations stimulated the development of HIV-PIs with molecular modifications. Then, second-generation HIV-PIs have been developed to treat HIV strains resistant to other HIV-PIs, and include darunavir (DRV) and atazanavir (ATV) [15]. In the treatment of HIV infection, DRV and ATV have demonstrated efficacy, safety, reduced toxicity, and low risk of regimen failure [19,20]. However, its antifungal properties have not yet been explored. Therefore, we evaluated the antifungal activity of the HIV-PIs darunavir (DRV) and atazanavir (ATV) on virulence factors of *C. albicans* and experimental candidiasis in the *Galleria mellonella* model.

## 2. Material and Methods

### 2.1. Microorganisms and Drugs

We used two clinical strains of *Candida albicans* isolated from oropharyngeal candidiasis lesions of patients diagnosed with AIDS and not yet treated: *C. albicans* 60 (Ca60) and *C. albicans* 70 (Ca70) strains [21]. A reference strain of *C. albicans* (ATCC 18804) was also included. These strains were cultured on Sabouraud dextrose agar (SDA; Difco, Detroit, USA) at 37 °C for 24 h before each assay. The HIV protease inhibitors used were darunavir (DRV; Sigma–Aldrich, St. Louis, Missouri, USA) and atazanavir (ATV; Sigma–Aldrich). The antifungal fluconazole (FCZ; Sigma–Aldrich) was also used in the experiments. The drugs were diluted in dimethyl sulfoxide (DMSO) and stored at −20 °C.

### 2.2. Susceptibility of C. albicans Planktonic Cells to HIV-PIs

To determine the susceptibility of *C. albicans* strains to DRV, ATV, and FCZ, we used the broth microdilution method proposed by the Clinical and Laboratory Standards Institute (CLSI) document M27-A2 [22]. Concentrations ranging from 1024 to 1 μg/mL were tested. *C. albicans* colonies were suspended in PBS, quantified using a hemocytometer, and diluted in Roswell Park Memorial Institute (RPMI) 1640 medium (Sigma–Aldrich) to a concentration of 1 × 10^3^ cells/mL. After the adjusted inoculum was added, the microplates were incubated at 37 °C for 48 h. The minimum inhibitory concentration (MIC) was visually determined as the lowest concentration capable of inhibiting *C. albicans* growth by 100%. *Candida albicans* ATCC 18804 was used as quality control. Sterility and growth controls were also included. The susceptibility assay was performed in duplicates.

### 2.3. Effect of HIV-PIs in Biofilm Formation of C. albicans

Suspension of the Ca60 and Ca70 strains were counted using a hemocytometer and adjusted to 10^7^ viable cells/mL. Initially, 100 μL of the standardized suspension was added to 96-well microplates containing Yeast Nitrogen Peptone (YNB; Difco) with 100 mM glucose and incubated for 90 min at 37 °C with stirring at 75 rpm for the initial adhesion phase. Subsequently, the supernatant was removed, each well was washed twice with PBS to remove non-adherent cells, 200 µL of fresh YNB containing DRV, ATV, and FCZ (3x MIC) was added to the plates, and incubated for 48 h at 37 °C with shaking. The medium was removed and fresh YNB was added after 24 h. A control group treated with PBS was used for each strain. After treatment, each well was washed twice with PBS for the subsequent analysis of cell viability and total biomass.

To evaluate the effect of HIV-PIs on biofilm viability, PBS was added to each well and the biofilms were disrupted using an ultrasonic homogenizer (Sonopuls HD 2200, Bandelin Electronic, Berlin, Germany) at 50 W for 30 s. The suspension was plated on SDA and the plates were incubated at 37 °C for 48 h to determine the number of colony-forming units (CFU/mL) [23].

Biofilm biomass was quantified by crystal violet staining [24]. Following this, the biofilm was fixed with methanol (Sigma–Aldrich), stained with a crystal violet 1% solution, washed with 0.85% NaCl, and discolored with peracetic acid. The supernatant was transferred to new 96-well microplates and the absorbance was measured at 570 nm (AJX-1900 spectrophotometer, Micronal, São Paulo, Brazil). Both assays were performed in triplicates.

### 2.4. Effect of HIV-PIs on C. albicans Filamentation

The effects of ATV and DRV on the filamentation of Ca60 and Ca70 strains were evaluated. In a 24-well culture plate, 2 mL of deionized water was mixed with 10% fetal bovine serum (FBS) (Sigma-Aldrich) and 100 μL of standardized *C. albicans* suspension (10^7^ viable cells/mL). DRV (3× MIC) and ATV (3× MIC) were also added to the experimental groups. For the control group, 50 μL of PBS was added to each well. The plates were incubated at 37 °C for 24 h (5% CO_2_). After incubation, 50 μL of the inoculum was transferred to glass slides with 10 previously demarcated fields on the back of the slide and observed under a light microscope at 400 × magnification in triplicates. Images were analyzed and 10 microscopic fields per slide were chosen for hyphal quantification. A score (0–5) was assigned to each field according to the number of hyphae observed as follows: 0, no hyphae; 1, 1–10 hyphae; 2, 11–20 hyphae; 3, 21–30 hyphae; 4, 31–40 hyphae; and 5, more than 41 hyphae [25].

### 2.5. Effect of HIV-PIs on SAP2 and BCR1 Gene Expression

Biofilms of the Ca70 strain were formed in 24-well microtiter plates, as previously described [26]. Subsequently, 1 mL TRIzol (Ambion, Carlsbad, CA, USA) was added to each well to extract RNA, according to the manufacturer’s protocol. RNA concentration was measured using a NanoDrop 2000 Spectrophotometer (Thermo Fisher Scientific, Wilmington, DE, USA). Total extracted RNA (700 ng) was treated with RQ1 RNase-Free DNase (Promega Corporation, Madison, WI, USA) and transcribed to cDNA using the GoScript Reverse Transcription Mix, Random Primers (Promega Corporation), according to the manufacturer’s recommendations. *Candida albicans BCR1* and *SAP2* primers were used [27,28]. cDNA was amplified to determine the relative quantification of *BCR1* and *SAP2* expression. The *RIP1* reference gene was tested in all experimental groups.

For quantitative polymerase chain reaction (qPCR) reactions, a GoTaq qPCR Master Mix kit (Promega Corporation) was used, according to the manufacturer’s recommendations. The reactions were performed in two biological replicates using the StepOnePlus Real-Time PCR System (Applied Biosystems, Foster, CA, USA). The 2^−ΔΔCT^ method was used to calculate the gene expression levels of *BCR1* and *SAP2* [29].

### 2.6. Evaluation of HIV-PI Treatment of Experimental Infection by C. albicans in Galleria Mellonella

*G. mellonella* larvae were maintained in the laboratory as described by Jorjão et al. [30], and larvae weighing ~330 mg without color alterations were selected. Ten randomly selected *G. mellonella* larvae were used per group for all the assays. Two controls were included: one group was inoculated with PBS and the second group, as control, received no injection to evaluate general viability. The larvae were pretreated by injecting 10 µL of DRV or ATV (20 mg/kg) through the last left proleg using a Hamilton 10 μL syringe. Suspensions of Ca60 and Ca70 strains were prepared from cultures in 5 mL of YPD liquid medium and incubated at 37 °C for 18 h. Cells were then centrifuged at 2000× *g* for 10 min and the supernatant was discarded. Cell pellets were dissolved in PBS and homogenized. This procedure was repeated twice. The cell densities were counted using a hemocytometer and adjusted to 10⁸ cells/mL. Two hours after treatment with ATV or DRV, the larvae were infected with 10^6^ cells/larva of *C. albicans* through the last right proleg (10 µL). Larvae were stored in Petri dishes at 37 °C and monitored daily for seven days to determine the survival curve.

### 2.7. Statistical Analysis

Student’s t-test was used to compare the CFU/mL results from the in vitro biofilm formation assay and gene expression. The scores obtained from the in vitro filamentation analysis were compared using the Kruskal–Wallis and Dunn’s tests. Percent survival and survival curves of *G. mellonella* were plotted and statistical analysis was performed using the log-rank test. All analyses were performed using GraphPad Prism 5.0 (GraphPad Inc., San Diego, CA, USA), and a *p* value ≤ 0.05 was considered significant.

## 3. Results

### 3.1. HIV-PIs Showed Antifungal Activity against C. albicans Planktonic Cells

Initially, we evaluated the susceptibility of planktonic cells to HIV-PIs and FCZ using reference and clinical strains of *C. albicans*. The minimum inhibitory concentration (MIC) of DRV and ATV were 512 µg/mL for *C. albicans* ATCC 18804, *C. albicans* 60 (Ca60), and *C. albicans* 70 (Ca70). The MIC of fluconazole (FCZ) ranged from 1 to >64 µg/mL for *C. albicans* strains (Table 1).

### 3.2. HIV-PIs Decreased Viability and Biomass of C. albicans Biofilms

**Figure 1:**
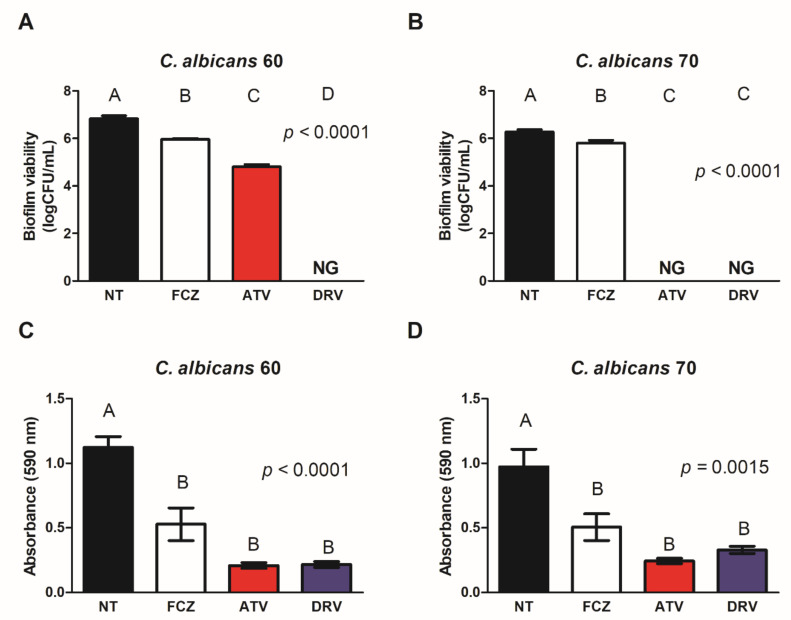
ATV and DRV reduced the viability and biomass of *Candida albicans* biofilms. Fungal viability of biofilms of (**A**) *C. albicans* 60 (Ca60) and (**B**) *C. albicans* 70 (Ca70) not treated (NT) and treated with fluconazole (FCZ), darunavir (DRV), and atazanavir (ATV). Biofilm biomass of (**C**) Ca60 and (**D**) Ca70 without treatment (NT) and after treatment with FCZ, DRV, and ATV. Results represent the mean of three replicates. FCZ: fluconazole; DRV: darunavir; ATV: atazanavir; NT = not treated; NG = no growth. Different letters (A, B, C, D) indicate significant differences (*p* < 0.05).

Based on the susceptibility of planktonic *C. albicans* cells to HIV-PIs, we evaluated the effect of these drugs (10× MIC value) on biofilms formed by Ca60 and Ca70 strains. HIV-PIs reduced the viability of both Ca60 and Ca70 strains. DRV (1536 μg/mL) decreased Ca60 viability (2, 27 log) and led to complete inhibition of Ca70 growth (Figure 1A,B). ATV (1536 μg/mL) completely inhibited fungal growth in both strains (Figure 1A,B). Treatment of biofilms with HIV-PIs also reduced fungal biomass. DRV (1536 μg/mL) reduced the biomass of Ca60 and Ca70 by 81 and 67%, respectively, whereas ATV (1536 μg/mL) decreased fungal biomass by 82% for both strains (Figure 1C,D).

### 3.3. HIV-PIs Inhibited the Filamentation of C. albicans

**Figure 2:**
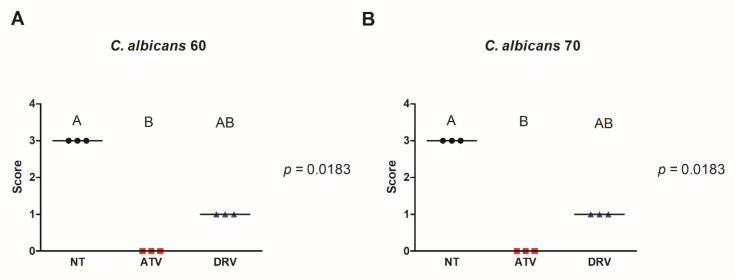
ATV and DRV decreased the number of *C. albicans* filaments. Scores attributed for hyphae formation of (**A**) *C. albicans* 60 (Ca60) and (**B**) *C. albicans* 70 (Ca70) not treated (NT) and treated with darunavir (DRV) and atazanavir (ATV). Results represent the mean of three replicates. DRV: darunavir; ATV: atazanavir; NT = not treated. Different letters (A, B) indicate significant differences (*p* < 0.05).

Since HIV-PIs reduced cell viability and biofilm biomass, we evaluated the influence of these drugs on *C. albicans* filamentation. ATV and DRV decreased the filamentation of both Ca60 and Ca70 strains and total inhibition of hyphal formation was observed after treatment with ATV (Figure 2).

### 3.4. HIV-PIs Downregulated the Expression of SAP2 and BRC1 in C. albicans

**Figure 3:**
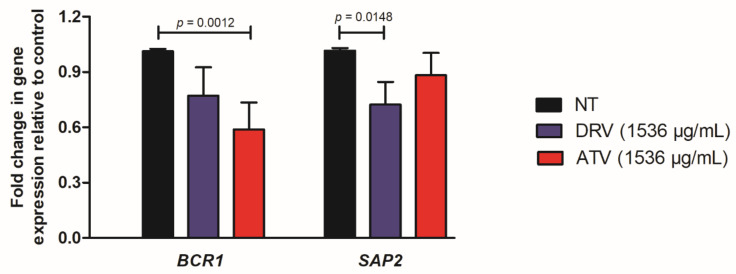
Relative expression of *C. albicans* genes. Relative quantification of *BCR1* and *SAP2* genes for Ca70 biofilms non-treated (NT) and treated with darunavir (DRV) and atazanavir (ATV). Normalization was performed using the *RIP1* gene. DRV and ATV treatments were compared with NT using the student’s *t*-test (*p* < 0.05).

To elucidate the possible effects involved in the anti-biofilm action of HIV-PIs, we quantified the expression of two genes related to biofilm formation (*BRC1*) and proteinase secretion (*SAP2*) in a fluconazole-resistant *C. albicans* strain (Ca70). The expression of *SAP2* was significantly downregulated by DRV treatment, while *BCR1* expression showed a significant downregulation by ATV treatment (Figure 3).

### 3.5. HIV-PIs Increased the Survival of G. mellonella Infected with C. albicans

**Figure 4:**
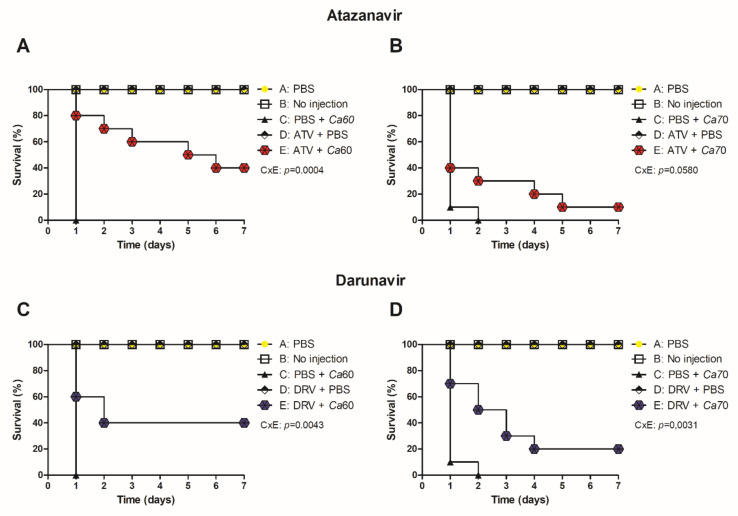
ATV and DRV increased the survival of *G. mellonella* infected with *C. albicans* strains. Survival curve of uninoculated *G. mellonella* larvae (no injection), infected with (**A**,**C**) *C. albicans* 60 and (**B**,**D**) *C. albicans* 70 and treated with PBS, atazanavir (ATV), or darunavir (DRV). Larvae were pre-treated with ATV or DRV at the last left proleg and infected with *C. albicans* at the last right proleg. Ca60: *C. albicans* 60; Ca70: *C. albicans* 70; DRV: darunavir; ATV: atazanavir. *p* < 0.05.

The antifungal effects of ATV and DRV determined in vitro were expanded to an in vivo model of *G. mellonella*. All uninfected larvae treated with PBS or HIV-PIs survived for seven days. Larvae infected with Ca60 or Ca70 strains and untreated larvae died between 24 and 48 h (Figure 4). In contrast, larvae treated with ATV and infected with the Ca60 and Ca70 strains increased the survival rate by 40 and 10%, respectively (Figure 4A,B). Concerning DRV, the treatment increased the survival of larvae infected with Ca60 by up to 40% and 20% for Ca70 (Figure 4C,D).

## 4. Discussion

The development of new therapeutic strategies against *Candida* infections is needed because of the limited arsenal of antifungal compounds, toxic effects, elevated costs, and the emergence of fungal strains resistant to currently available drugs. However, the processes that involve prospecting, screening, and marketing new antifungals are expensive and time-consuming [31]. Therefore, drug repositioning assumes an important role when investigating new uses for existing drugs and has emerged as an interesting and promising tool against fungal infections [14,32]. Thus, HIV-PIs have gained special attention due to their antimicrobial activity in different fungal species, including *C. albicans* [11,18,33]. In this study, we evaluated the activity of the second-generation HIV-PIs DRV and ATV against *C. albicans*. To the best of our knowledge, this is the first study with a focus on DRV and ATV as a therapy for *C. albicans* and candidiasis.

We found that ATV and DRV exhibited antifungal activity against planktonic cells of *C. albicans* at 512 μg/mL. In previous studies, DRV MIC values ranging from 256 to 512 μg/mL were found for other fungal species, such as *Cryptococcus neoformans* and *C. gattii* [18]. Another second-generation HIV-PI, lopinavir, exhibited MIC values greater than 128 μg/mL for *Candida auris*, an emerging multi-drug resistant yeast [34]. Taken together, these studies indicated the necessity to use a high MIC value of HIV-PIs as monotherapy for inhibiting the growth of fungal cells. These data can suggest the use of HIV-PIs as adjuvants to conventional antifungal drugs. Brilhante et al. [34] demonstrated that DRV had a synergistic effect with amphotericin B against *Cryptococcus* species. In combined treatment, the MIC values of both DRV and amphotericin B were reduced in comparison to monotherapy. These authors hypothesized that the cellular membrane alterations caused by amphotericin B may have facilitated the penetration of a greater amount of antiretroviral in the cytoplasm of *Cryptococcus* cells. Therefore, future studies of HIV-PIs ATV and DRV against *Candida* species should be focused on their association with amphotericin B, azoles, or echinocandins.

To understand the action of ATV and DRV on fungal virulence factors, we evaluated the role of HIV-PIs in the biofilm formation and filamentation of *C. albicans*. *Candida* biofilms are important because of their ability to colonize biotic and abiotic surfaces and their reduced susceptibility to antifungal therapy. This reduced susceptibility is due to intrinsic factors in its structure, such as the difficulty of drug diffusion into the extracellular polysaccharide matrix and the expression of genes related to efflux pumps, which can interfere with the response to different drugs. The difficulty in treating biofilms requires an increase in antifungal doses, causing toxicity, which reinforces the limitations of the therapeutic arsenal available [35,36].

Here, ATV and DRV exhibited anti-biofilm effects by reducing biomass and cell viability post-treatment. Previous studies demonstrated the ability of ATV to disrupt mature *C. albicans* biofilms in association with caspofungin. In contrast, ATV was ineffective in preventing *C. albicans* biofilm formation [11]. The anti-biofilm action of HIV-PIs amprenavir and lopinavir in monotherapy was demonstrated for *C. albicans* [9,10]. Brilhante et al. [18] also found a decrease in the biomass and metabolic activity of *Cryptococcus gattii* and *C. neoformans* biofilms after treatment with DRV.

In this study, we also evaluated the effect of ATV and DRV treatments on the filamentation of *C. albicans*, another virulence factor important for fungal pathogenicity. *C. albicans* hyphae and pseudohyphae are invasive forms capable of penetrating the host tissue, causing tissue damage and eventually organ colonization [37]. Therefore, filamentation represents an attractive target for developing therapeutic approaches against candidiasis [38]. We demonstrated that treatment with DRV reduced and ATV completely inhibited *C. albicans* filamentation, reinforcing the anti-biofilm effect of these HIV-PIs. Using scanning electron microscopy, Santos et al. [9] demonstrated that although treatment with HIV-PI lopinavir did not fully inhibit *C. albicans* filamentation, the remaining yeast cells exhibited significant surface injuries associated with decreased fungal viability.

The reduction in *C. albicans* filamentation, biomass, and biofilm viability was associated with the downregulation of *BRC1* and *SAP2* genes observed in ATV and DRV treatment. The importance of *SAP* in the pathogenicity of *C. albicans* is well established. Infection with *C. albicans* mutants for the *SAP1*, *SAP2*, and *SAP3* genes resulted in decreased mortality in mice [39] and reduced tissue damage in the human reconstituted epithelium [40]. Previous studies exploring the relationship between treatment with HIV-PIs and the activity of SAPs demonstrated that lopinavir, saquinavir, indinavir, and amprenavir blocked the hydrolytic activity of the Sap1, Sap2, and Sap3 enzymes [9,10,41]. In addition, indinavir, tipranavir, and ritonavir were potent inhibitors of Sap production in *C. albicans* culture [12,42,43], while amprenavir and indinavir reduced the expression of Sap1, Sap2, and Sap3 antigens bound to the yeast cell wall [10,44].

Thus, our study is the first to evaluate ATV and DRV action in *SAP* expression in *C. albicans* biofilms, demonstrating the inhibitory activity of these HIV-PIs on the gene encoding Sap2, an important extracellular protease in the Saps family. These results are promising since some studies have shown that SAPs play a multimodal role in the morphogenesis, adhesion, and biofilm development of *C. albicans*, and their inhibition directly affects fungal growth and viability. Furthermore, several studies have demonstrated the importance of *SAP2* overexpression in azole-resistant *C. albicans* strains [10,12]. Here, we observed that DRV treatment reduced *SAP2* expression in biofilms of *C. albicans* 70 (Ca70), a fluconazole-resistant strain.

Likewise, Ca70 biofilms treated with HIV-PIs exhibited downregulation in *BRC1* expression, especially with ATV. The transcription factor bcr1 plays a prominent role in regulating *C. albicans* filamentation and biofilm formation [45,46,47]. *C. albicans* strains with mutations in the bcr1 alleles were unable to form biofilms and establish the initial adhesion phase. In addition, other studies have demonstrated the participation of this factor in cell filamentation in the opaque state [45]. Thus, ATV and DRV reduced filamentation, biofilm viability, and biomass, and downregulated the expression of genes involved in *C. albicans* virulence.

Based on the in vitro results, the *G. mellonella* model was used to evaluate the protective effects of ATV and DRV against experimental candidiasis. Interestingly, ATV and DRV increased the survival of *G. mellonella* compared with the untreated group. Previous studies demonstrated that the combination of lopinavir and itraconazole increased the survival of the invertebrate *Caenorhabditis elegans* (90%) infected with *C. auris* AR0390. In contrast, lopinavir did not increase larval survival in monotherapy [11]. Combination treatment with darunavir and ritonavir in HIV-positive patients elevated immune system function, characterized by a progressive increase in lymphocyte proliferation in response to *Candida* until reaching normal levels after 48 weeks of therapy [48]. These in vivo data reinforce the antifungal action of HIV-PIs in a manner associated with an improvement in the immune status, which may explain the increased survival of *G. mellonella*. Further studies are required to confirm this hypothesis.

Finally, it is important to emphasize that our results are limited to a few *Candida* strains analyzed. Only three *C. albicans* strains were investigated, including one reference strain and two clinical strains obtained from non-treated patients diagnosed with AIDS. It is known that there are genetic variations among *Candida* strains that implicate different profiles of virulence and susceptibility to antimicrobial agents. Therefore, further trials with other *C. albicans* isolates and non-*albicans* strains, mainly multidrug-resistant strains, are required to strengthen the anti-*Candida* activity of HIV-PIs.

In summary, the second-generation HIV-PIs DRV and ATV exhibited in vitro antifungal activity against *C. albicans* in both planktonic and biofilm stages. Furthermore, ATV and DRV reduced filamentation and the expression of virulence-related genes in *C. albicans*, associated with a protective effect in experimental candidiasis in *G. mellonella*. Importantly, HIV-PIs also showed inhibitory effects against a fluconazole-resistant strain, suggesting that ATV and DRV warrant more attention in future studies of drug repositioning strategies for *Candida* infections.

## Figures and Tables

**Table 1:** Minimal inhibitory concentration values (MIC; µg/mL) of atazanavir (ATV), darunavir (DRV), and fluconazole (FCZ) against *C. albicans* strains ATCC 18804, *C. albicans* 60 and *C. albicans* 70.

Strains	MIC (µg/mL)		
	Darunavir (DTV)	Atazanavir (ATV)	Fluconazole (FCZ)
*C. albicans* ATCC 18804	512	512	2
*C. albicans* 60	512	512	1
*C. albicans* 70	512	512	>64
Range	512	512	1 to >64

## Data Availability

Not applicable.
